# Carotidynia: A Rare Diagnosis for Unilateral Neck Pain Revealed by Cross-Sectional Imaging

**DOI:** 10.1155/2017/7086854

**Published:** 2017-09-24

**Authors:** Corrado Santarosa, Salvatore Stefanelli, Roman Sztajzel, Pravin Mundada, Minerva Becker

**Affiliations:** ^1^Division of Radiology, Department of Imaging and Medical Informatics, Geneva University Hospitals, Geneva, Switzerland; ^2^Clinic of Neurology, Department of Clinical Neurosciences, Geneva University Hospitals, Geneva, Switzerland

## Abstract

Idiopathic carotidynia (IC) is a rare and poorly understood syndrome consisting of unilateral neck pain, tenderness, and increased pulsations over the affected carotid bifurcation. A growing body of evidence supports the hypothesis that IC is a distinct clinicopathologic entity with characteristic imaging features. We report the case of a 34-year-old Caucasian male presenting with intense unilateral neck pain in the emergency setting. Computed tomography and ultrasonography revealed fusiform eccentric thickening of the ipsilateral carotid bifurcation without vessel narrowing. Contrast-enhanced magnetic resonance imaging depicted major perivascular enhancement without evidence of dissection. Further imaging and laboratory work-up excluded vasculitis. The diagnosis of IC was made. The patient was treated with nonsteroidal anti-inflammatory drugs and symptoms and imaging findings disappeared within a few weeks. Cross-sectional imaging allows not only ruling out IC mimickers but also making the correct diagnosis of this rare condition, in particular, as the clinical presentation of IC is often nonspecific.

## 1. Introduction

Idiopathic carotidynia (IC) is a controversial clinical entity first reported by Fay in 1927 [1]. According to the diagnostic criteria of the first International Classification of Headache Disorders (ICHD) in 1988, IC was described as unilateral self-limiting neck pain with tenderness, swelling, and increased pulsations over the affected carotid bifurcation and without structural vascular abnormality [2]. As authors reporting this entity did not rule out other causes of unilateral neck pain, it was suggested that IC may not be a distinct disease [3]. Therefore, in 2004, IC was removed from the ICHD classification and was considered merely a pain syndrome [4]. However, in the following years, isolated IC cases with perivascular inflammation depicted by cross-sectional imaging were reported.

We herewith present a case of IC with characteristic clinical and cross-sectional imaging features, further supporting the hypothesis that IC is most likely a distinct inflammatory disease of the carotid bifurcation.

## 2. Case Report

A 34-year-old male presented to the emergency department complaining of bitemporal headache for 5 days and left neck pain for 24 hours irradiating into the ipsilateral mandibular and auricular region. The neck pain was intense, throbbing, continuous, and localized over the left carotid bifurcation. It increased with neck movements and upon palpation. Physical and neurological examination was normal. No trauma, infection, or chiropractic neck manipulation was reported. The patient's medical history was otherwise unremarkable. Laboratory tests such as peripheral blood cell and platelet count, hemoglobin, hematocrit, prothrombin and partial thromboplastin time, sodium, potassium, glucose, creatinine, urea, and liver function tests were all normal; in particular the white cell blood count was of 7 g/l (reference range: 4–10 g/l) and the C-reactive protein (CRP) was 6.3 mg/l (reference range: 0−10 mg/l). The erythrocyte sedimentation rate was the only laboratory test just slightly above the upper reference limit (11 mm/h; reference range: 0–10 mm/h).

**Figure 1:**
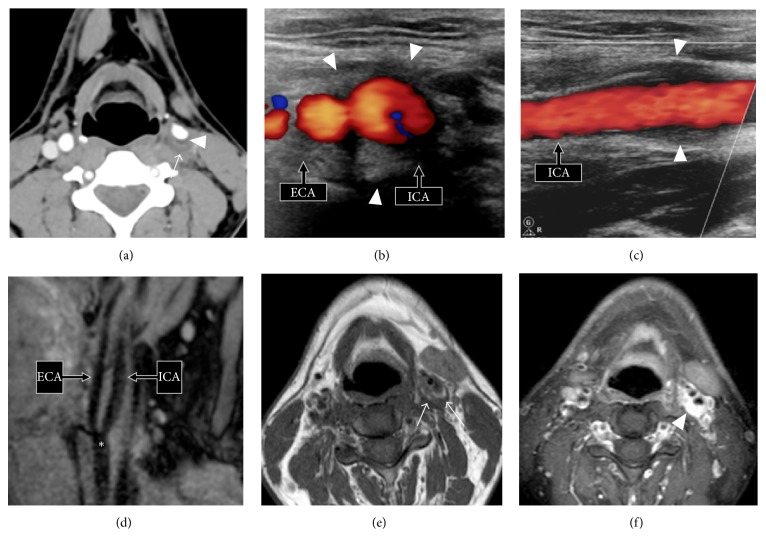
(a) Axial contrast-enhanced CT scan (soft tissue window) shows poorly delineated thickening of the left carotid bulb wall* (arrow)*. Note obliteration of the pericarotid fatty tissue. Minimal vessel narrowing (<20%) due to fibrolipid plaque* (arrowhead)*. (b, c) Axial and longitudinal views of Doppler US examination show fusiform eccentric wall thickening of the left proximal internal carotid artery (ICA) and external carotid artery (ECA) isoechoic to muscle* (arrowheads)*. (d) Sagittal multiplanar reformatted (MPR) image from a 3D volume turbo spin echo acquisition centered on the left carotid bulb* (asterisk)* shows no evidence of spontaneously T1 hyperintense parietal hematoma, thus ruling out carotid artery dissection. No aneurysm is seen. (e) Axial T1-weighted MR image reveals a hypointense circumferential lesion involving the left carotid bulb and pericarotid fatty tissue* (arrows)*. (f) Intense enhancement* (arrowhead)* of the mass-like soft tissue lesion on the axial postcontrast fat-saturated T1-weighted MR image. Note that perivascular enhancement and vessel wall enhancement can be hardly differentiated from one another. ICA: internal carotid artery and ECA: external carotid artery.

Contrast-enhanced computed tomography (CT) and Doppler ultrasonography (US) were obtained. CT showed soft tissue thickening surrounding the distal common carotid artery, the carotid bulb, and the proximal internal carotid artery on the affected side (Figure 1). No pathology of the other supra-aortic arteries or of the aortic cross was found. At Doppler US, the thickened arterial wall was isoechoic to muscle and peak systolic velocity was 90 cm/s. The internal vessel aspect was normal (Figure 1). No arterial blood flow turbulence or flow velocity acceleration was observed. No intimal dissection or aneurysm was seen. Contralateral carotid arteries were unremarkable. Although the examinations were not designed to evaluate neck mass, no obvious neck mass or lymphadenopathy was seen.

The patient underwent an additional contrast-enhanced magnetic resonance imaging (MRI) scan the following day to rule out dissection. It showed eccentric soft tissue thickening of the left carotid bifurcation with hypointense signal on the T1-weighted sequence and hyperintense signal on the T2-weighted sequence. No T1 hyperintense parietal carotid hematoma was seen on the fat-saturated T1-weighted sequence (Figure 1). After intravenous administration of contrast material, intense enhancement encasing the carotid bifurcation was present suggesting an inflammatory etiology.

**Figure 2:**
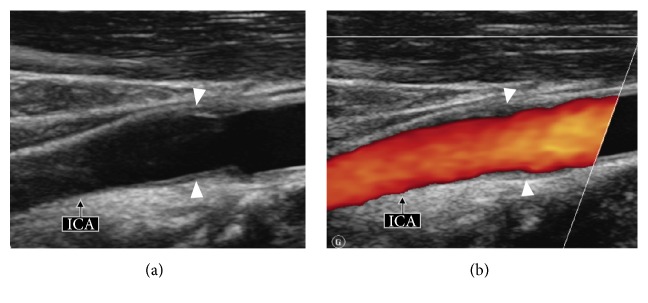
US longitudinal view (a) and corresponding color Doppler image (b) obtained 4 months later show regression of the fusiform wall thickening (arrowheads) of the left carotid bulb. ICA: internal carotid artery. See for comparison Figures 1(b) and 1(c) obtained 4 months earlier.

After negative immunological laboratory tests including anti-neutrophil cytoplasmic antibodies (ANCA), antinuclear antibodies (ANA), and anti-nucleoprotein (RNP-Sm-SSA-SSBScl70-Jo1) antibodies, as well as an abdominal MRI showing no vascular abnormality (no fibromuscular dysplasia, no vasculitis, and no stenosis), the diagnosis of IC was established and the patient was treated with nonsteroidal anti-inflammatory drugs (NSAIDs). Treatment with NSAIDs resulted in disappearance of neck pain within a few weeks. Follow-up Doppler US obtained 4 months later (Figure 2) revealed disappearance of the abnormal soft tissue lesion surrounding the affected carotid bifurcation. The patient continued to be asymptomatic 6 months later.

## 3. Discussion

IC is a controversial and rare diagnostic entity [5–9]. The mean age of presentation is 37 years (range: 15–78 years) [9]. Most cases are unilateral; however, up to 10% of cases are bilateral [9]. Patients usually present with throbbing neck pain, occasionally irradiating into the ear and worsening during swallowing, during mastication, or while turning the head. Increased pulsations over the affected carotid bifurcation and pain exacerbation upon palpation are common [8, 9]. Although currently the ICHD does not recognize IC as a separate disease but rather considers IC as a syndrome of variable etiology, there is increasing evidence suggesting that IC is most likely a distinct clinicopathologic entity. IC has a benign course with spontaneous pain resolution within a few weeks (most often <2 weeks). A common treatment option to expedite symptom resolution is the administration of NSAIDs or corticoids.

The etiology of IC is not known. A case of IC probably induced by fluoxetine (Prozac) has been reported in a patient with recurrent episodes of migraine and mild depression who developed IC during treatment with fluoxetine, remitted on fluoxetine discontinuation, and relapsed after drug challenge [10]. Other IC cases have been described in patients undergoing chemotherapy for Burkitt lymphoma and in patients affected by infectious conditions, such as latent tuberculosis or laryngopharyngeal infection [11–13]. However, a causal relationship between the underlying condition and IC could not be proven [11, 12].

Upton et al. and Farage et al. reported results of surgical biopsy in patients with IC [14, 15]. Biopsy specimens of pericarotid tissues and carotid adventitia revealed nonspecific chronic inflammation with proliferation of fibroblasts, lymphocytes, mastocytes, and eosinophils, without granuloma formation and without evidence of active infection [14, 15]. Proliferation of small vessels embedded in a fibromyxoid stroma was characteristic. These inflammatory changes were distinct from histologic findings in large vessels involved by vasculitis, and no giant cells were present at histopathology [14]. Based on the above-mentioned histologic features, it was suggested that IC may represent a distinct form of carotid/pericarotid inflammation, vasculitis, or even an inflammatory pseudotumor of the carotid space [14, 15]. Furthermore, some authors suggested that levels of CRP, serum amyloid protein, fibrin degradation product D-dimer, and soluble intracellular adhesion molecule-1 appear to correlate with the clinical stage of carotidynia, thus further supporting the hypothesis of an inflammatory etiology [16, 17]. Unlike published case reports, the inflammatory markers were within normal limits in our patient; in particular we found no evidence of vasculitis despite extensive imaging and laboratory work-up.

Similar to other case reports, the patient in the current report displayed an infiltrative pericarotid mass-like soft tissue lesion without arterial narrowing. Nevertheless, some authors reported that substantial carotid narrowing may occur in the presence of extensive vessel encasement [14]. The infiltrative soft tissue masses surrounding the carotid bifurcation most often show intense enhancement on MRI and differentiation between the enhancing abnormal pericarotid soft tissues and the carotid wall itself is hardly possible. The enhancing soft tissue masses often have smooth margins thus suggesting containment within the carotid sheet fascia. Although the lack of structural vessel abnormality was one of the main criteria for the diagnosis of IC in the first version of the ICHD [2], the characteristic imaging pattern increasingly reported in IC establishes—on the contrary—the central role of imaging for the diagnosis of IC.

Cross-sectional imaging also plays a major role in diagnosing other vascular conditions manifesting with unilateral neck pain and mimicking IC, such as arterial dissection, large vessel arteritis, arteriosclerosis, fibromuscular dysplasia, or aneurysms. The main differential diagnostic considerations at cross-sectional imaging include carotid dissection and large vessel arteritis. Carotid dissection can be ruled out at MRI due to the absence of a T1 hyperintense wall hematoma. When dissection occurs, a high signal intensity wall hematoma can be demonstrated by MRI up to several months after the dissection event. In large vessel arteritis (giant cell arteritis and Takayasu arteritis), the thickened arterial wall enhances intensively on MRI; however, this enhancement is generally associated with significant luminal stenosis and it is not limited to the carotid bifurcation.

## 4. Conclusion

IC is a benign self-limiting neck pain syndrome associated with inflammatory abnormalities on the affected carotid bifurcation, which can be elegantly demonstrated by cross-sectional imaging. Typically, there is only minimal or absent arterial luminal narrowing and soft tissue abnormalities disappear on follow-up studies. Imaging findings in conjunction with the clinical presentation not only enable a correct diagnosis and meaningful management of IC but equally rule out other diagnostic entities, which may have a similar clinical presentation.
